# Trans-arterial chemo-embolization (TACE), with either lipiodol (traditional TACE) or drug-eluting microspheres (precision TACE, pTACE) in the treatment of hepatocellular carcinoma: efficacy and safety results from a large mono-institutional analysis

**DOI:** 10.1186/1756-9966-29-164

**Published:** 2010-12-15

**Authors:** Mario Scartozzi, Gianluca Svegliati Baroni, Luca Faloppi, Marzia Di Pietro Paolo, Chiara Pierantoni, Roberto Candelari, Rossana Berardi, Stefania Antognoli, Cinzia Mincarelli, Andrea Risaliti, Cristina Marmorale, Ettore Antico, Antonio Benedetti, Stefano Cascinu

**Affiliations:** 1 Clinica di Oncologia Medica, AO Ospedali Riuniti-Università Politecnica delle Marche, via Conca, 60020, Ancona, Italy; 2 Clinica di Gastroenterologia, AO Ospedali Riuniti-Università Politecnica delle Marche, via Conca, 60020, Ancona, Italy; 3 Scuola di Specializzazione in Oncologia, Università Politecnica delle Marche, via Conca, 60020, Ancona, Italy; 4 Oncologia Medica, Ospedale Profili, Fabriano, Italy; 5 Radiologia Interventistica, AO Ospedali Riuniti, via Conca, 60020, Ancona, Italy; 6 Chirurgia Epatobiliare e dei Trapianti, AO Ospedali Riuniti-Università Politecnica delle Marche, via Conca, 60020, Ancona, Italy; 7 Clinica Chirurgica, AO Ospedali Riuniti-Università Politecnica delle Marche, via Conca, 60020, Ancona, Italy

## Abstract

More data about TACE and pTACE seem necessary to better define the global treatment strategy for HCC. Aim of our analysis was to evaluate the role of TACE, either with lipiodol (traditional) or drug-eluting microspheres in terms of response rate (RR), time to progression (TTP), overall survival (OS) and toxicity in HCC.

Patients with HCC undergoing traditional TACE or pTACE (either alone or in combination with other treatment options) were eligible

One hundred and fifty patients were analyzed. In the global patient population median OS was 46 months for lipiodol TACE and 19 months for pTACE (p < 0.0001), TTP was 30 months versus 16 months for patients receiving TACE or pTACE respectively (p = 0.003). These results were confirmed also among the group of patients who received exclusive TACE or pTACE. Neither RR nor toxicity was different between TACE or pTACE.

At multivariate analysis, age, the Okuda stage, type of TACE and number of TACE proved to be independent prognostic factors influencing overall survival.

In our experience, lipiodol TACE showed a better OS and TTP over pTACE, without difference in toxicity profile and RR. Among the staging systems analyzed only the Okuda stage seemed able to reliably predict patients outcome.

## Background

Hepatocellular carcinoma (HCC) represents the commonest primary cancer of the liver. Incidence is increasing and HCC has risen to become the 5th commonest malignancy worldwide and the third leading cause of cancer related death, exceeded only by cancers of the lung and stomach [1,2].

Surgery is the only potentially curative treatment for HCC. In carefully selected patients, resection and transplantation allow in fact a survival ranging from 60% to 70%, and should be considered as the preferred treatment options in early-stage disease with the assessment of hepatic functional reserve being essential for treatment planning [3].

The percutaneous treatment for HCC, percutaneous alcohol injection (PEI) and the radiofrequency thermal ablation (RF), are an alternative to surgery in patients with early stage disease who are not candidates to resection or transplantation [4,5].

The majority of patients in Western countries presents an intermediate or advanced stage at diagnosis. These patients are therefore candidates treatment including transarterial embolization and chemoembolization and systemic treatments including chemotherapy, immunotherapy and hormonal therapy [6]. Only recently, a molecular targeted drug, Sorafenib, has been proved effective in these patients [7-9].

TACE represents a crucial treatment option for HCC, however comparative assessment of clinical findings resulted often hampered by the considerable variability in patients selection criteria and modalities of execution of therapy [10-12]. Nonetheless meta-analyses of clinical trials suggested a favorable impact of this procedure on survival [13,14] and the reports of Lo and Llovet independently showed a significant increase in survival in patients treated with TACE compared to control group [15,16].

In the last few years pTACE (precision TACE with drug-eluting microspheres) presented as a possible further improvement in the treatment of HCC, but few data are available about its role, particularly in comparison with traditional TACE, for the global treatment strategy in HCC patients.

Primary aim of our analysis was to evaluate the role of transarterial chemoembolization, either with lipiodol (traditional TACE) or drug-eluting microspheres (precision TACE, pTACE), in terms of response rate (RR), time to progression (TTP) and overall survival (OS), in patients with advanced HCC.

Secondary aim of the study was to evaluate the role of pTACE compared to TACE and toxicity deriving from treatment.

## Materials and methods

### Patients selection

We have retrospectively analyzed a population of HCC patients, treated with TACE (lipiodol or drug-eluting microspheres) from 2002 to 2009, at our institution. The study included all patients consecutively treated with TACE (in our institution, patients were treated with TACE with lipiodol from 2002 until 2006 and with TACE with microspheres from 2007 to 2009).

All patients studied were suffering by liver cirrhosis, 70% on viral etiology (HBV and HCV chronic hepatitis), 15% on toxic etiology (alcohol), 15% caused by genetic and metabolic diseases.

Patients were divided into two groups. The first group included patients who received, as the sole treatment for HCC, either traditional TACE (selective TACE with infusion of chemotherapeutic agents associated with lipiodol, without the use of microspheres) or pTACE (superselective TACE with drug-eluting microspheres). The second group included patients who received TACE or pTACE in addiction to other treatments, such as liver resection, liver transplantation, alcoholic or laser ablation, radiofrequency thermal ablation, systemic therapies. Furthermore, we analyzed, separately the group of patients treated with traditional TACE or pTACE.

Patients were classified according to ECOG performance status and were staged using different staging systems to assess patients general clinical condition, extent of disease and liver function: TNM, Child-Pugh, CLIP, BCLC, Okuda, JIS, MELD, MELD-Na.

For each patient the dose of chemotherapy of each treatment were recorded, and the dose to the first treatment and the cumulative dose were assessed. Patients were then divided into two groups (high and low dose) in relation to the median dose of drug.

### Clinical outcome evaluation and statistical analysis

Treatment response was assessed through CT and MRI, α-FP assay, performed after one month of treatment and then every 3 months, according to the new RECIST criteria (New Response Evaluation Criteria in Solid Tumors 1.1). Radiological images were reviewed in double-blind by two radiologists.

The distribution curves of survival and time to progression were estimated using the Kaplan-Meier method. Overall survival (OS) was calculated as the time interval between the date of radiological or histological diagnosis of HCC and the date of death or last follow-up. The time to progression (TTP) was calculated as the time interval between the date of the traditional TACE or pTACE and the date of progression or last follow-up. Treatment toxicity was evaluated according to NCI-CTC 3.0 (National Cancer Institute - Common Toxicity Criteria 3.0). Toxicity profiles were grouped by severity (G1-G2 vs. G3-G4) and the time (early <1 week vs delayed >1 week)

The clinical variables analyzed were: gender (male vs. female), age (≤69 years vs. >69 years), ECOG performance status (0-1 vs. 2-3), TNM stage (I-IIIB vs IIIC - IV), the Child-Pugh score (A vs. B), the CLIP stage (0-1 vs >1), BCLC stage (A vs. B-C), Okuda stage (I vs. II vs. III), stage JIS (0-1 vs >1), the MELD score (≤10 vs. 11-15 vs. >15), the MELD-Na score (≤10 vs. 11-15 vs. >15), exclusive TACE vs. TACE + other treatments, the type of TACE (traditional TACE with lipiodol vs. pTACE with drug-eluting microspheres) and the number of re-treatments (1 vs. 2 vs. ≥3).

The association between variables was estimated using the chi-square test.

The Cox multiple regression analysis was used for those variables that were found significant at the univariate analysis.

Any differences between the groups were considered significant if the significance level was less than 0.05.

## Results

One hundred and fifty patients were available for our analysis: 122 (81%) males and 28 (19%) females. Median age was 69 years (range 49-89) (Table 1).

**Table 1:** Patients characteristics and main results.

Patients	General series	TACE exclusive	TACE non exclusive	TACE exclusive lipiodol	TACE exclusive microspheres
	n = 150	n = 82	n = 68	n = 50	n = 32
**Median Age (range)**	69 (40-89)	72 (41-89)	66 (40-84)	74 (42-89)	68 (41-79)
**OS months (range)**	32 (3-124)	30 (3-91)	32 (3-124)	46 (3-87)	14 (3-91)
**TTP months (range)**	24 (1-64)	26 (1-64)	24 (1-52)	32 (1-64)	13 (1-28)
					
**Gender (%)**					
					
male	122 (81)	65 (79)	57 (84)	36 (79)	29 (91)
female	28 (19)	17 (21)	11 (16)	14 (21)	3 (9)
**Patients undergoing TACE (%)**					
					
TACE exclusive	82 (55)				
TACE non exclusive	68 (45)				
**Type of TACE (%)**					
					
TACE	87 (58)	50 (61)	37 (54)		
pTACE	63 (42)	32 (39)	31 (46)		
**OS months (Type of TACE) (range)**					
					
TACE	46 (3-124)				
pTACE	19 (3-91)				
**TTP months (Type of TACE) (range)**					
					
TACE	30 (1-64)				
pTACE	16 (1-38)				

Eighty-two patients (55%) received TACE or pTACE as the only therapeutic approach, while 68 patients (45%) received also other treatments.

In the group of patients treated with TACE only, 50 (61%) underwent traditional TACE, while 32 (39%) received pTACE with microspheres.

All groups of patients showed similar clinical characteristics according to all staging systems used (Table 2).

**Table 2:** Patients sub-groups stratification according to staging systems used in our analysis

Staging systems						
**Patients**		**General series**	**TACE exclusive**	**TACE non exclusive**	**TACE exclusive lipiodol**	**TACE exclusive microspheres**
**Score (%)**		**n = 150**	**n = 82**	**n = 68**	**n = 50**	**n = 32**

ECOG	0-1	133 (89)	73 (89)	60 (88)	42 (84)	31 (97)
	2-4	17 (11)	9 (11)	8 (12)	8 (16)	1 (3)
TNM	1-3B	130 (87)	72 (88)	58 (85)	44 (88)	28 (87)
	3C-4	20 (13)	10 (12)	10 (15)	6 (12)	4 (13)
Child-Pugh	A	87 (58)	39 (48)	48 (70)	26 (51)	14 (43)
	B	63 (42)	43 (52)	20 (30)	24 (49)	18 (57)
CLIP	0-1	92 (61)	47 (57)	44 (64)	29 (58)	18 (57)
	> 1	58 (39)	35 (43)	24 (36)	21 (42)	14 (43)
BCLC	A	74 (46)	32 (39)	41 (61)	19 (38)	13 (41)
	B-C	76 (54)	50 (61)	27 (39)	31 (62)	19 (59)
Okuda	1	98 (65)	50 (61)	47 (69)	31 (62)	19(60)
	2	48 (32)	27 (33)	21 (31)	17 (33)	11 (33)
	3	4 (3)	5 (6)	0 (0)	2 (5)	2 (7)
JIS	0-1	79 (52)	37 (45)	41 (60)	24 (49)	13 (40)
	> 1	71 (48)	45 (55)	27 (40)	26 (51)	19 (60)
MELD	≤10	101 (67)	53 (65)	49 (72)	32 (64)	21 (67)
	11-15	42 (28)	25 (30)	18 (27)	15 (30)	9 (29)
	> 15	7 (5)	4 (5)	1 (1)	3 (6)	2 (4)
MELD-Na	≤10	65 (43)	37 (45)	39 (57)	21 (42)	16 (50)
	11-15	58 (39)	37 (45)	22 (33)	22 (45)	15 (46)
	> 15	27 (18)	8 (10)	7 (10)	7 (13)	1 (4)

In the whole group, median survival was 32 months, while median time to progression was 24 months. Patients treated with TACE only showed a median survival of 30 months, compared to 32 months for patients treated with other treatments in addition to TACE (p = 0.69). The time to progression was 26 months versus 24 months respectively in patients treated with TACE only and in those treated with other therapies (p = 0.85).

**Figure 1:**
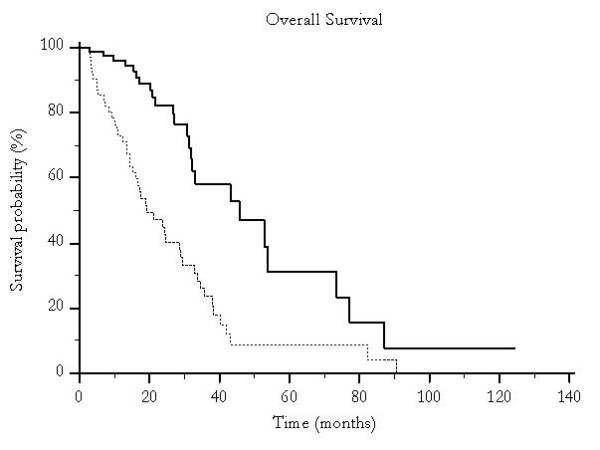
**Median overall survival for patients undergoing traditional TACE (---) and for those who were treated with pTACE (---------**) **(46 vs 19 months, p < 0.0001)**.

**Figure 2:**
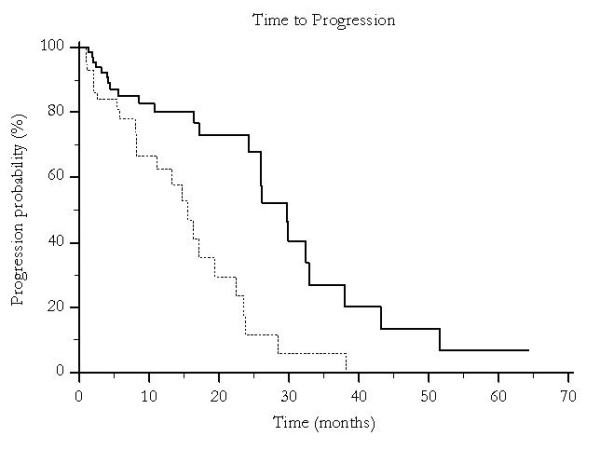
**Median time to progression for patients undergoing traditional TACE (---) and for those who were treated with pTACE (---------) (30 vs 16 months, p < 0.003)**.

**Figure 3:**
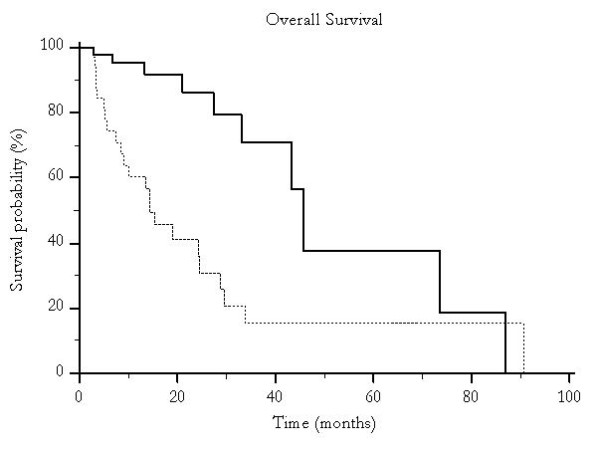
**Median overall survival for patients undergoing traditional TACE (---) and for those who were treated with pTACE (---------) (46 vs 14 months, p = 0.0002)**. Only patients receiving exclusive TACE were considered.

**Figure 4:**
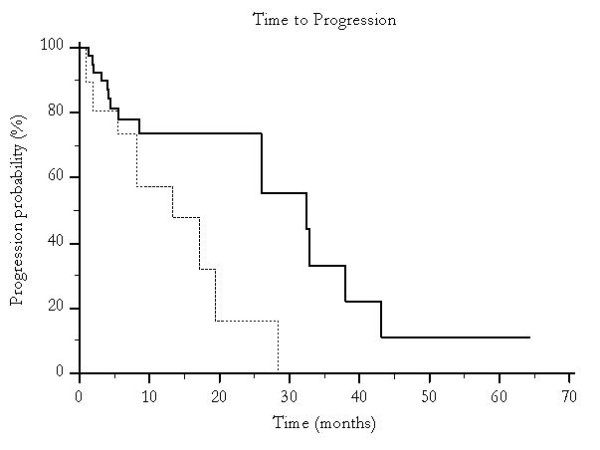
**Median time to progression for patients undergoing traditional TACE (---) and for those who were treated with pTACE (---------) (32 vs 13 months, p = 0.014)**. Only patients receiving exclusive TACE were considered.

Median overall survival was 46 months for patients undergoing traditional TACE and 19 months for those who were treated with pTACE (p < 0.0001) (Figure 1) and time to progression was 30 months versus 16 months for patients receiving either traditional TACE or pTACE respectively (p = 0.003) (Figure 2). These results were confirmed also among the group of patients who received exclusive traditional TACE or pTACE as the only treatment approach. In particular median overall survival was 46 months for patients treated with lipiodol TACE compared to 14 months for patients treated with pTACE (p = 0.0002) (Figure 3). Median time to progression was 32 months for patients treated with traditional TACE compared to 13 months for patients treated with pTACE (p = 0.014) (Figure 4).

**Figure 5:**
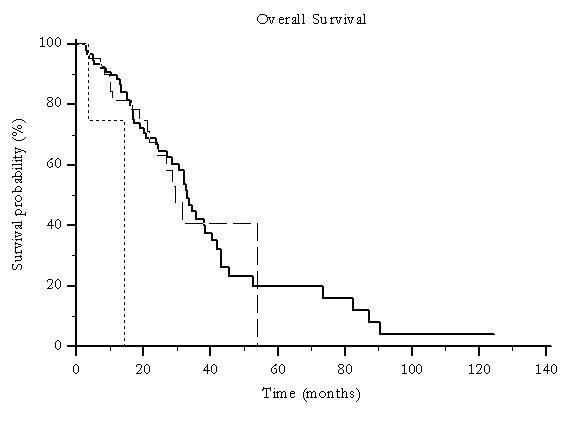
**Median overall survival for global patients population according to the Okuda staging system: Okuda 1(---), Okuda 2 (---------) and Okuda 3 (.........) (33 vs 29 vs 14 months, p = 0.046)**.

At the univariate analysis, age (p < 0.0001), Okuda stage (p = 0.046) (Figure 5), type of TACE (P < 0,0001) and number of TACE treatments (p = 0.003) were found to be prognostic factors influencing overall survival. Type of TACE (p = 0.0003) and the number of TACE treatments (p = 0.004) were also found to be prognostic factors influencing the time to progression.

At multivariate analysis, age, the Okuda stage, type of TACE and number of TACE treatments proved to be independent prognostic factors influencing overall survival (p < 0.0001). Only type and number of TACE treatments proved to be independent prognostic factors influencing time to progression (p < 0.0001).

Overall response rate for patients treated with lipiodol TACE or pTACE respectively was: complete response in 17 (20%) and 14 (24%) patients, partial remission in 32 (39%) and 19 (33%) patients, stable disease in 16 (19%) and 7 (12%) patients, and progressive disease in 18 (22%) and 18 (31%) patients.

No statistically significant differences in terms of objective response (assessed according to RECIST criteria) was found between the groups of patients treated with lipiodol TACE or pTACE with microspheres (Table 3).

**Table 3:** Response rate observed in the global case series and according to treatment received (lipiodol TACE or pTACE) (CR = complete remission; PR = partial remission; SD = stable disease; PD = progressive disease NA = not available)

Objective response			
	**TACE lipiodol**	**pTACE microspheres**	**Total**
	
**CR (%)**	17 (20)	14 (24)	31 (22)
			
**PR (%)**	32 (39)	19 (33)	51 (36)
			
**SD (%)**	16 (19)	7 (12)	23 (15)
			
**PD (%)**	18 (22)	18 (31)	36 (27)
			
**NA**	8	1	9

The toxicity profiles (were not statistically different between the groups of patients treated with lipiodol TACE or pTACE (Table 4).

**Table 4:** Main toxicity results for lipiodol TACE and pTACE according to NCI-CTC 3.0 (National Cancer Institute - Common Toxicity Criteria 3.0).

Toxicity								
	**Total**				**G3 - G4**			
	**TACE lipiodol**		**pTACE microspheres**		**TACE lipiodol**		**pTACE microspheres**	
	**early**	**late**	**early**	**late**	**early**	**late**	**early**	**late**

**Hepatic (%)**								
transaminase	31 (41)	6 (8)	22 (33)	11 (16)	7 (9)	-	2 (3)	-
γ-gt	22 (29)	9 (12)	16 (24)	12 (18)	5 (7)	3 (4)	5 (7)	6 (9)
alkaline phosphatase	8 (11)	4 (5)	7 (10)	7 (10)	-	-	-	-
bilirubin	25 (33)	2 (3)	16 (24)	5 (7)	3 (4)	2 (3)	3 (4)	-
coagulation	-	1 (1)	-	-	-	-	-	-
albumin	7 (9)	2 (3)	3 (4)	1 (1)	-	-	1 (1)	-
								
**Hematologic (%)**								

leukopenia	4 (5)	2 (3)	2 (3)	4 (6)	1 (1)	-	1 (1)	1 (1)
anemia	8 (11)	6 (8)	6 (9)	4 (6)	1 (1)	-	-	-
piastinopenia	22 (29)	6 (8)	21 (31)	11 (16)	1 (1)	-	6 (9)	2 (3)
								
**Other (%)**								

	pain	2 (5)	-	8 (23)	-	-	-5 (14)	
	fever	2 (5)	1 (3)	3 (9)	-	-	-	-

**Figure 6:**
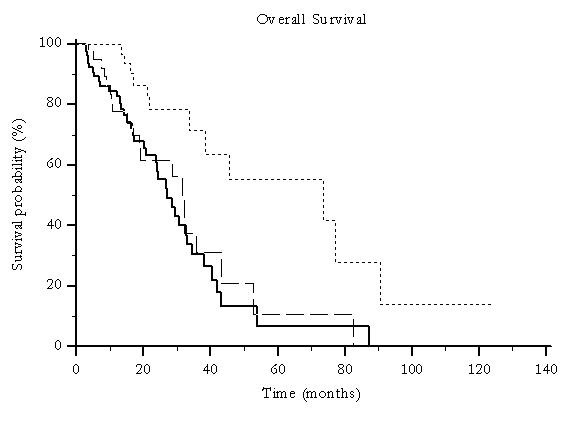
**Median overall survival for global patients population according to the number of TACE treatments delivered: 1TACE treatment (---), 2 TACE treatments (---------) and ≥ 3 TACE treatments (.........) (74 vs 31 vs 27 months, p = 0.0029)**.

**Figure 7:**
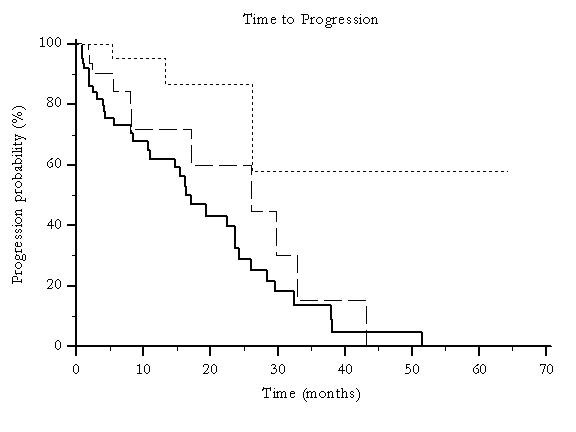
**Median time to progression for global patients population according to the number of TACE treatments delivered: 1TACE treatment (---), 2TACE treatments (--------) and ≥ 3 TACE treatments (.........) (p = 0.0042)**.

In the overall series, 32 (21%) patients underwent a minimum of 3 TACE treatments, 39 (26%) underwent 2 treatments and 79 (53%) received a single treatment. In these groups a statistically significant difference was noted for overall survival (p = 0.003) (Figure 6) and time to progression (p = 0.0042) (Figure 7). No correlations could be noticed between the number of treatments performed, stage of disease and liver function.

Fifteen (19%) patients who received traditional TACE or pTACE only were treated with at least 3 TACE sessions and showed a median survival of 74 months, 24 (29%) received 2 treatments with a median survival of 29 months (range 3-43) and 43 (52%) were subjected to a single treatment with a survival of 25 months (range 3-87) (p = 0.0286). The difference in time to progression was not statistically significant (p = 0.057).

In the whole patients population statistically significant differences were noted in relation to the dose of chemotherapy administered (< 53 mg or ≥53 mg) at the time of the first TACE or pTACE, for both median overall survival (46 months, vs 24 months, p < 0.0001) and time to progression (30 months vs 17 months, p = 0.0061).

## Discussion

Several studies have demonstrated the efficacy of TACE with lipiodol, for the treatment of HCC. However comparative assessment of results is often hampered by the considerable variability in patients selection criteria and in modalities of treatment administration.

Favorable results on overall survival for treatments with lipiodol TACE, reported by retrospective studies were initially questioned by randomized controlled clinical trials with groups of patients treated conservatively [10-12] with subsequent meta-analyses of previous clinical trials suggesting a favorable impact of this procedure on survival [13,14].

More recently the reports of Lo and Llovet independently showed a significant survival improvement for patients treated with TACE compared to control groups [15,16].

These results are probably attributable to the stringent criteria for patient selection and to the maintenance of results over time through repetition of the procedure, with an average of 2.8 TACE treatment per patient.

In the last years the treatment of pTACE with microspheres is increasingly arguing for the management of patients with HCC and recent studies have validated the effectiveness of pTACE with microspheres, in terms of objective response rate [17].

Two recent trials presented at the American Society of Clinical Oncology annual Meeting 2009, one retrospective [18], and one prospective [19] have shown an advantage in terms of overall survival and objective complete responses in favor of pTACE with microspheres for patients with unresectable HCC.

In our experience treatment with microspheres could not confirm these findings, in particular for overall survival and time to progression. On the contrary in our series median overall survival resulted improved in the group of patients treated with lipiodol TACE compared to the group of patients treated with microspheres, while no significant differences were noticed in terms of response rate.

Although these apparently conflicting results may be related to the retrospective nature of our study, differences in the patients population investigated and to inevitable selection bias, we should note that the sample size analyzed in the present study is considerably larger than the sample size presented in the analog retrospective trial by Dhanasekaran et al.

The enrollment time itself (11 years in the study by Dhanasekaran vs 7 years in our analysis) could have influenced results as well, with the longer enrollment time in the trials by Dhanasekaran possibly putting at stake sample homogeneity.

Unfortunately the trial by Lencioni et al does not include information about overall survival and time to progression, but only data about response rate., which resulted improved for pTACE. Nevertheless although not significant in our study response rate for TACE and pTACE are comparable to those reported by Lencioni, thus suggesting an effective reproducibility of our results in the clinical practice.

It is possible that pTACE with microspheres could have a greater embolizant effect than TACE with lipiodol, and this would lead to increased tumor growth factors release in response to hypoxia, with consequently probability of recurrence and reduced overall survival and time to progression. The response rate, assessed at one month after treatment, however, is similar between the two groups, because these molecular mechanisms would not be able to influence it, resulting in a statistically significant difference in such a short time. In this setting treatment with sorafenib may represent a valuable asset to further improve clinical results.

Our analysis also showed a more pronounced treatment benefit for older patients. This observation may be related to either a more aggressive tumor behavior in younger patients or a more indolent tumor progression in older age (or to a combination of both considerations).

Many patients in our series received more sessions of TACE or pTACE treatments during their medical history. These patients seem to have obtained an advantage in terms of overall survival and time to progression compared to those treated with a single TACE or pTACE session. This seems to imply that certain biological characteristics could make certain HCC more or less responsive to treatment with TACE. These considerations should of course be considerate merely speculative.

Further studies focusing on biological and clinical characteristics of HCC should be conducted before definitive conclusion could be drawn.

The observation that patients who received a sub-median dose of drug may have an advantage in terms of overall survival and time to progression compared to those who received a dose over-the median deserves further comments. It is possible that a higher dose of chemotherapy would result in an additional damage to a liver function already heavily compromised due to the underlying disease, rather than an advantage, measurable with a tumor shrinkage.

Another crucial point of discussion in HCC is the use of a staging system which effectively reproducible.

In our study none of the staging systems commonly used in clinical practice has proven to be able to classify patients from a prognostic point of view, with the exception of the Okuda system, which proved able to influence the overall survival (p = 0.046).

Unlike most other malignancies, for which the staging systems are well codified and universally accepted the staging systems proposed for HCC are not universally adopted and shared. One of the reasons that makes it difficult to obtain reliable results, is related to the fact that in most cases, the tumor occurs in patients with liver cirrhosis. Therefore tumor stage, liver function and clinical characteristics may differently concur to define subgroups of HCC in different patients.

In this perspective, the results of our analysis proved to agree with the majority of studies in the literature.

## Conclusion

The clinical management of HCC is becoming increasingly complex as therapeutic options are expanding. The patient has, in most cases, two diseases, cancer and the underlying liver disease that often heavily influenced, by mechanisms not yet completely clear, the response to cancer therapy and prognosis. So it is clear how crucial is a multi-specialist management of patients with HCC.

In this framework, loco-regional treatment still plays an important role and appears to be an essential point of comparison even, and maybe even more, in the era of biological therapies.

## Abbreviations

(TACE): Transarterial chemoembolization; (traditional TACE): TACE with lipiodol; (precision TACE, pTACE): TACE with drug-eluting microspheres; (RR): response rate; (TTP): time to progression; (OS): overall survival; (HCC): hepatocellular carcinoma; (PEI): percutaneous alcohol injection; (RF): radiofrequency thermal ablation.

## Competing interests

The authors declare that they have no competing interests.

## Authors' contributions

MS: conception, design, analysis and interpretation of data, revising the manuscript. GSB: conception and design. LF: conception, design, acquisition analysis and interpretation of data, writing of the manuscript. MDPP: acquisition analysis and interpretation of data. CP: acquisition analysis and interpretation of data. RC: acquisition of data. RB: acquisition analysis and interpretation of data. SA: acquisition analysis and interpretation of data. CM: acquisition of data. AR, CM, EA, and AB: revised the study. SC: conception, design, analysis and interpretation of data, revising the study. All authors read and approved the final manuscript.
